# Mechanosensing regulates virulence in *Escherichia coli* O157:H7

**DOI:** 10.1080/19490976.2015.1121365

**Published:** 2016-03-03

**Authors:** Md. Shahidul Islam, Anne Marie Krachler

**Affiliations:** a Department of Biotechnology, Bangladesh Agricultural University, Mymensingh, Bangladesh; b Institute of Microbiology and Infection, School of Biosciences, University of Birmingham, Edgbaston, Birmingham, United Kingdom

**Keywords:** attaching/effacing pathogens, enterohemorrhagic *Escherichia coli*, gastrointestinal infection, host-pathogen interactions, locus of enterocyte effacement, mechanosensing

## Abstract

Enterohemorrhagic *Escherichia coli* O157:H7 is a food-borne pathogen transmitted via the fecal-oral route, and can cause bloody diarrhea and hemolytic uremic syndrome (HUS) in the human host. Although a range of colonization factors, Shiga toxins and a type III secretion system (T3SS) all contribute to disease development, the locus of enterocyte effacement (LEE) encoded T3SS is responsible for the formation of lesions in the intestinal tract. While a variety of chemical cues in the host environment are known to up-regulate LEE expression, we recently demonstrated that changes in physical forces at the site of attachment are required for localized, full induction of the system and thus spatial regulation of virulence in the intestinal tract. Here, we discuss our findings in the light of other recent studies describing mechanosensing of the host and force-dependent induction of virulence mechanisms. We discuss potential mechanisms of mechanosensing and mechanotransduction, and the level of conservation across bacterial species.

*Escherichia coli* O157:H7 (enterohemorrhagic *E. coli*, or EHEC) is a food-borne pathogen and can cause bloody diarrhea and sometimes hemolytic uremic syndrome (HUS) in humans. While the diarrhea is usually self-limiting and resolves over the course of several days, HUS is a severe complication which can lead to lasting kidney damage, and is associated with high morbidity and mortality.^1^ EHEC is taken up via the fecal-oral route and, once inside the human host, it colonizes the large intestine and initiates a virulence program leading to the above described pathophysiology. EHEC's virulence arsenal includes adhesins, a type 3 secretion system (T3SS), and Shiga toxins, which all contribute to disease.^2-4^ The action of the T3SS, which is encoded by a pathogenicity island termed locus of enterocyte effacement (LEE), is responsible for the formation of characteristic attaching and effacing lesions in the intestine, and contributes to disease severity.^5,6^ The formation of A/E lesions roughly corresponds to the formation of actin protrusions, termed pedestals, in tissue culture models of infection, and this has allowed a more thorough investigation of this phenotype. The LEE-encoded T3SS translocates effector proteins into the host cell cytoplasm, where they modulate host cellular signaling to facilitate host colonization, immune modulation, and bacterial persistence.^7^ Most notably, their actions result in cytoskeletal rearrangements, pedestal formation, and stable anchoring of the bacterium to the host cell, although their effects are more wide-ranging and effector repertoire and activities are still subject to ongoing studies.

The LEE pathogenicity island is a large region encompassing more than 40 open reading frames, organized into 5 major transcriptional units (LEE1-5), and has been horizontally acquired. Its expression underlies global, H-NS mediated silencing outside the host, where its costly-to-produce gene products are not beneficial for survival.^8^ Once inside the host, EHEC senses the change in environment through a change in temperature and a range of chemical cues, and gradually adjusts its expression profile as it passes through the GI tract, in a way that poises the organism to colonize the large intestine, where it specifically initiates expression of LEE in a highly site-specific manner. Over the years, many groups have added to our knowledge about the nature of different environmental signals that contribute to LEE induction, and about the genetic elements integrating them. Many of these studies were done in other A/E-pathogens, most notably enteropathogenic *E. coli* (EPEC), which also encode the LEE and are similarly, although not identically, regulated as the EHEC LEE.^9^ All known activation processes proceed via LEE-encoded regulator (Ler), the first product encoded by LEE1 and the master regulator for the entire LEE (Fig. 1). Ler acts as an antirepressor that counteracts H-NS mediated silencing by displacing H-NS from a nucleoprotein complex around the promoter regions within the LEE.^8^ Expression of Ler, in turn, is regulated by a number of upstream activators, which may differ in their nature between different strains and include BipA, PchABC, IHF, and QseA, among others. Arguably the most important of these activators is the global regulator of Ler (GrlA), which unlike other regulators, is directly encoded within the LEE. GrlA is a MerR like transcription activator, which acts by locally unwinding the DNA and optimizing the spacing between the Ler promoter −10 and −35 elements.^10^ GrlA is expressed from a transcriptional unit together with GrlR, which is able to bind to and inhibit GrlA, and this is thought to be an important regulatory mechanism of GrlA activity.^11,12^ A number of environmental cues which trigger activation of Ler have been identified, including human body temperature, low oxygen, neutral pH, and the presence of bicarbonate and quorum sensing autoinducers, among others. ^13-16^ While these mechanisms point toward a gradual enhancement in LEE expression directly after passage through the acidic stomach and further, upon contact with bicarbonate upon entry into the small intestine. Additional studies suggest a further level of fine-tuning in LEE expression through the sensing of human hormones^17^, and through the site specific composition of the intestinal microbiota. *Bacteroides thetaiotamicron* (*B. theta*), a commensal of the lower GI tract, provides cues for LEE induction by generating fucose through cleavage from mucins in the large intestine.^18^

**Figure 1.**
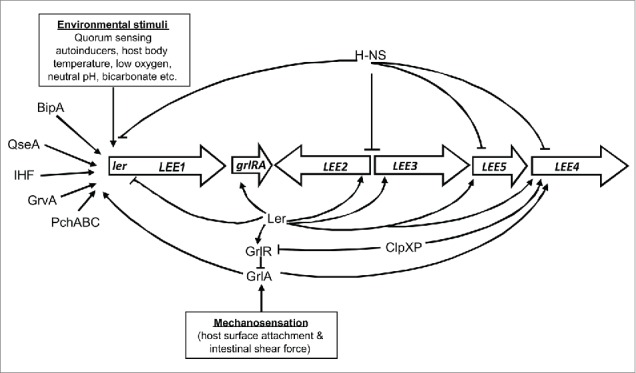
Activation of locus of enterocyte effacement (LEE) genes in enterohemorrhagic *E. coli* O157:H7. Outside the host, LEE genes are silenced by the global repressor H-NS. Once inside the host, different environmental stimuli and transcription factors partially activate LEE genes through induction of Ler expression (Ler antagonizes H-NS repression). Mechanosensation causes complete activation of LEE genes through the full induction of Ler in a GrlA - dependent manner. Transcriptional activators and repressors are shown by pointed and blunt arrows, respectively. Figure adapted from Kendall et al.^28^

Our recent studies of LEE induction in a tissue culture infection model add a further layer of complexity to this existing picture. We show, by using enzymatic and fluorescent reporters of Ler induction, that LEE expression, albeit weakly induced upon contact with known environmental cues (such as glucose present in the host medium, and elevated temperature of 37°C), is only fully induced upon direct physical contact with the host cell surface.^19^ This induction of Ler proceeds via the action of GrlA. However, our results change the perspective on the role GrlR plays in repressing GrlA mediated LEE activation. Since it was previously shown that GrlR forms a tight complex with GrlA^12^, thereby preventing its access to the Ler promoter, it was assumed that GrlR was sufficient to repress GrlA, and that any mechanism activating GrlA would proceed by disrupting the inhibitory GrlRA complex. Our results however demonstrate that, while GrlR is inhibitory to GrlA activity, free GrlA is not fully functional in activating LEE expression per-se, but requires further cues (i.e., host cell contact), to fully activate LEE. The mechanism behind the transition in GrlA to become fully functional is unclear, and a number of scenarios are conceivable. Host cell contact could either lead to recruitment of another, yet unknown, factor which could increase GrlA's affinity for the Ler promoter. Alternatively, it could result in biochemical and/or structural changes in GrlA which could facilitate its promoter binding. Further, contact sensing could result in a change in GrlA subcellular localization, which could facilitate its access to the promoter. Further experiments to test these scenarios are currently underway. We further show, using a range of pure substrates, that this induction does not require a specific ligand-receptor interaction, but instead is dependent on strong attachment to a surface. Attached cells are even further induced by application of shear forces, as demonstrated through cells immobilized in microfluidic flow cells and exposed to increasing amounts of laminar fluid flow. The level of promoter induction scales both with strength of adhesion and the applied shear force. In EHEC bound to host cells, the induction level saturates at shear forces of approximately 1 dyne/cm^2^, which is within the physiological range of shear force likely prevalent in the intestine. Although this is challenging to evaluate experimentally, hydrodynamic calculations of shear forces in the intestinal tract estimate the fluid shear on the luminal surface at approximately 5 dynes/cm^2^, and between 2-3 dynes/cm^2^ between microvili.^20^

Our experiments indicate that EHEC directly senses physical force and can integrate information about different types of forces (here, surface adhesion and shear force) to achieve gene regulation. While chemical cues partially induce the LEE and poise the bacterium for binding by low-level expression of factors necessary for strong attachment, mechanosensing triggers full induction of LEE expression directly at the site of infection. These findings raise many further, exciting questions about the way EHEC and other bacteria can perceive not only their chemical, but also their mechanical environment. The first question pertains to the nature of forces bacteria can sense. While our experiments demonstrate EHEC's ability to sense both adhesion and shear forces (which act perpendicular and parallel to the cell wall, respectively), there are many other forces bacteria are exposed to and could potentially perceive as environmental cues. Most notably, EHEC has to transition from the gut lumen and through the mucus layers, to reach the intestinal epithelial surface. This transition is accompanied by a marked change in viscosity. This will impact flagellar load, as well as cause an increase in shear force. Flagella have been implicated as mechanosensors across different bacterial species, usually in the context of inanimate surface sensing. *Bacillus subtilis*, for example, uses inhibition of flagellar rotation as a cue for surface contact, and induces biofilm formation in response.^21^ In *B. subtilis*, this response in gene expression is mediated via the DegS-DegU 2-component system, but how exactly the mechanical trigger activates this system has yet to be determined. While in *B. subtilis*, surface sensing appears to promote a global switch in gene expression toward a sessile life-style, mechanosensing via polar flagella have also been linked to the induction of virulence-specific genes. *Vibrio parahaemolyticus*, a sea-food borne pathogen which possesses a dual flagellar system, a decrease in flagellar rotation triggers the synthesis of lateral flagella necessary for surface motility, as well as expression of genes required for colonization and pathogenesis in the host.^22,23^ Interestingly, albeit a similar role in mechanosensing for purported for the *V. cholerae* flagellum, this was subsequently disproved.^24^

Recent work on *Pseudomonas aeruginosa* has revealed an alternative mode of surface sensing and mechano-induction of a virulence program in response to host cell contact. Attachment of *P. aeruginosa* to the amoebic model host *Dictyostelium discoideum* or to mouse macrophages was shown to increase cytotoxicity toward host cells, compared to planktonic bacteria.^25^ Further work by the same group showed that in *P. aeruginosa*, mechanoperception is mediated by type IV pili, and their changed ability to extend and retract following surface attachment.^26^ In *P. aeruginosa*, pilus retraction under physical tension upon surface attachment is thought to lead to a structural change in PilA pilus subunits, which facilitates an interaction with and activation of the transmembrane protein PilJ. PilJ activates the chemosensory complex ChpA-PilI, which then stimulates the adenylate cyclase CyaB and leads to cAMP production. cAMP activates the cAMP binding transcription factor Vfr, thereby increasing the transcription of virulence genes.^26^ These studies provide first mechanistic insights into how mechanosensing and mechanotransduction can be linked, although many details remain to be investigated. Although it is attractive to speculate mechanotransduction pathways are conserved across species, this is unlikely in the case of *P. aeruginosa* and *E. coli*. While *P. aeruginosa* PilJ bears high sequence identity with *E. coli* methyl-accepting chemotaxis proteins (MCPs), it has no direct homolog in *E. coli*. This suggests the mechanotransduction pathways linking force perception at the cell surface and gene regulation in the cytoplasm, are not strictly conserved between these 2 organisms. However, *E. coli* also has type IV pili ^27^ and it is conceivable that they may act as mechanosensors, as may other appendages, such as flagella.

In conclusion, our and other groups’ recent work has highlighted a role for mechanosensing in the induction of virulence-specific programmes in a range of bacterial pathogens. While technical advances over the past years, such as the commercialization of controlled flow systems, and their experimental combination with high-content imaging, has made it possible to investigate the effect of defined physical forces on gene expression, this has brought forward many important questions, which remain to be addressed. How are several different forces integrated to impact gene expression? What is the nature of bacterial mechanosensors and mechanotransduction pathways, and to what extent are they conserved across species? Addressing these questions in future studies will further extend this exciting area of research, but may also highlight new targets in our search for novel treatments against bacterial infections.
